# G2/M Checkpoint Dependency in High-Grade Serous Tubo-Ovarian Carcinoma: Mechanisms and Therapeutic Implications

**DOI:** 10.3390/genes17091135

**Published:** 2026-09-17

**Authors:** Zhiyu Tian, Karen McLean

**Affiliations:** 1 Department of Pharmacology & Therapeutics, Roswell Park Comprehensive Cancer Center, Buffalo, NY 14203, USA; zhiyu.tian@roswellpark.org; 2 Department of Gynecologic Oncology, Roswell Park Comprehensive Cancer Center, Buffalo, NY 14203, USA

**Keywords:** ovarian cancer, HGSC, G2/M checkpoint, cyclin, WEE1, CDK1, ATM, ATR

## Abstract

High-grade serous carcinoma (HGSC) of tubo-ovarian origin is an aggressive malignancy in need of new treatment options. At the molecular level, HGSC is characterized by genomic instability, including nearly universal *TP53* mutations, copy number alterations, and chromothripsis, which elevate replication stress. These fundamental alterations result in tumor cell dependence on the G2/M checkpoint for survival, thus revealing this checkpoint as a potential therapeutic target. In this review, we discuss the normal cell cycle progression and checkpoints as well as the regulation of DNA replication, and we consider how replication stress in HGSC leads to a dependence on the G2/M checkpoint. We then present information on the current state of targeted therapy development for HGSC including clinical trials and the role of biomarkers in optimal patient selection. Thus, targeting the G2/M checkpoint is driven by sound molecular rationale and may yield clinical benefit in patients with HGSC, but will likely require careful patient selection and consideration of treatment toxicities.

## 1. Introduction

High-grade serous carcinoma (HGSC) is the most common and aggressive subtype of ovarian cancer [1]. The current treatment approach for HGSC typically includes both surgery and chemotherapy, most commonly with a platinum agent plus a taxane. While the disease is initially responsive to therapy, the majority of HGSC recur and ultimately develop platinum resistance, requiring other systemic therapy approaches. Thus, new treatment approaches are needed. As discussed below, the underlying genetics and molecular alterations in HGSC provide unique targeted therapy approaches for clinical translation. This review focuses on the mechanistic rationale and therapeutic potential of targeting the G2/M checkpoint of the cell cycle in HGSC. We then present the mechanistic rationale for therapeutic vulnerabilities and discuss current inhibitors currently in clinical trial.

The cell cycle is a sequence of ordered transitions through four phases: G1 (growth 1), S (synthesis), G2 (growth 2), and M (mitosis). Normal cell cycle progression is regulated by cyclin-dependent kinases (CDKs) in response to upstream checkpoint signaling pathways. The G1/S transition in normal cells is regulated by CDK4/6-cyclin D complexes that phosphorylate the retinoblastoma protein (RB) to relieve RB-mediated suppression of E2 promoter-binding factor (E2F)-dependent transcription and allowing activation of CDK2-cyclin E [2].

During the G1 and S phases, DNA replication is a tightly regulated process and requires coordinated origin licensing, origin firing, DNA unwinding, and DNA synthesis [3]. During the G1 phase, replication origins are licensed through the loading of replication machinery onto DNA, ensuring that each origin is prepared for replication. Then, in the S phase, helicases unwind DNA and DNA polymerases synthesize new DNA strands at replication forks [4], the active sites where parental DNA is unwound and copied into new DNA strands. Replication stress refers to conditions that slow or stall replication fork progression or prevent accurate DNA synthesis [5]. Replication stress occurs by multiple mechanisms as discussed in this review. Furthermore, when replication fork integrity is compromised, unresolved DNA damage can accumulate and activate checkpoint signaling pathways [5].

**Figure 1:**
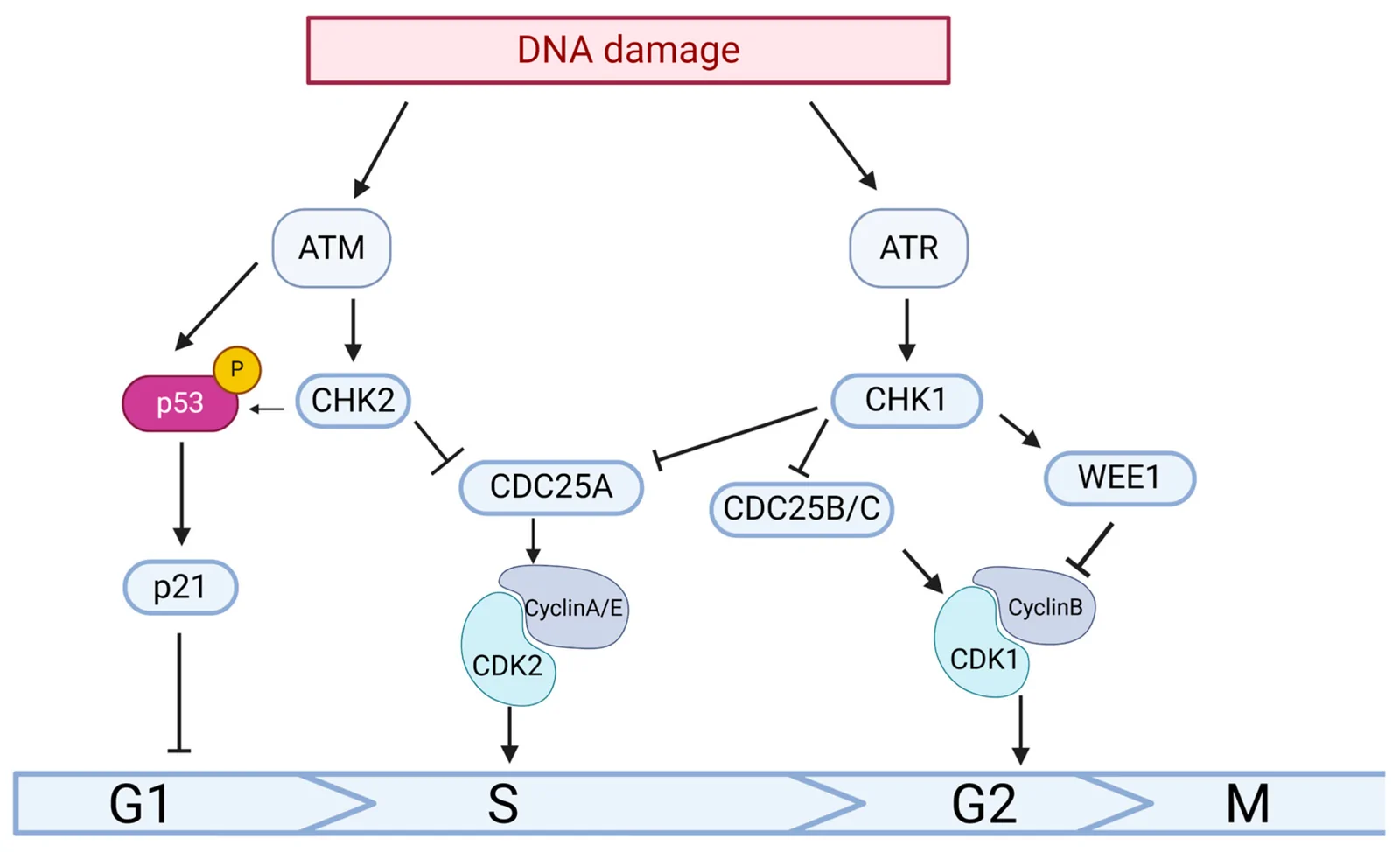
**Checkpoint regulations under normal conditions.** In response to DNA damage, ATM activates CHK2 and promotes p53 phosphorylation, leading to p21 induction and inhibition of G1/S progression. CHK2 can also suppress CDC25A, limiting CDK2 activity and slowing S phase entry. ATR activates CHK1, which regulates the intra-S and G2/M checkpoints by suppressing CDC25 phosphatases and maintaining WEE1-mediated inhibitory phosphorylation of CDK1-cyclin B, keeping CDK1 activity restrained and delaying mitotic entry until DNA repair and replication are complete.

If DNA double strand breaks are detected, the Ataxia-telangiectasia mutated (ATM) protein is activated and signals through checkpoint kinase 2 (CHK2) and p53 to promote p21 induction (Figure 1) [6], which inhibits CDK2 activity and can also restrain CDK4/6 activity, thereby inducing G1/S cell cycle arrest to allow time for DNA repair before proceeding to mitosis [7,8]. HGSC is characterized by almost universal mutations in the *TP53* gene that encodes the p53 tumor suppressor [9,10]. In *TP53*-mutated tumors, the G1 checkpoint is impaired, permitting cells with unresolved DNA damage to enter the S phase [6,11]. As a result, replication forks are more likely to encounter unrepaired DNA damage during the S phase, collapse, and generate additional DNA damage, leading to further elevated replication stress and the need for G2/M checkpoint to halt further cell cycle progression.

At the molecular level, replication stress is sensed when stalled replication forks generate stretches of single strand DNA. This single strand DNA is coated by replication protein A (RPA), which promotes recruitment and activation of the ataxia telangiectasia and Rad3-related (ATR)-ATR-interacting protein (ATRIP) complex. Activated ATR then signals through CHK1 to regulate the intra-S and G2/M checkpoints (Figure 1) [12]. A functional G2/M checkpoint allows accurate replication and once checkpoint signaling is resolved, CDK1-cyclin B activation drives entry into mitosis (Figure 1). Thus, HGSC cells become highly dependent on the ATR-CHK1-WEE1 axis which regulates the intra-S and G2/M checkpoints (Figure 1) [6]. Replication stress activates ATR-CHK1 signaling, resulting in suppression of CDC25 phosphatases and WEE1-mediated inhibitory phosphorylation of CDK1 within the CDK1-cyclin B complex. The suppression of this complex then delays mitotic entry to prevent cells from entering mitosis with DNA that is incompletely replicated or damaged.

In normal cells, checkpoints act as surveillance mechanisms that delay cell cycle progression when DNA damage, incomplete replication, or other cellular stresses are detected. With intact responses, premature entry into mitosis results in mitotic catastrophe and cell death [13,14]. In cancer, deregulation of CDK activity and checkpoint control can allow cells to proliferate despite genomic damage, contributing to uncontrolled growth [15].

Thus, in tumors with an impaired G1 checkpoint, these stresses increase reliance on G2/M checkpoint regulation for tumor survival. This dependency creates an opportunity for targeted therapy. Inhibition of key proteins in these pathways can disrupt checkpoint signaling and force cells into premature mitosis, ultimately leading to mitotic catastrophe and cancer cell death. This rationale also supports combination strategies with platinum agents, poly (ADP-ribose) polymerase (PARP) inhibitors, or other therapies that induce DNA damage. In this review, we discuss the molecular basis of replication stress-associated G2/M checkpoint dependency in HGSC and highlight therapeutic strategies that exploit this vulnerability.

## 2. Replication Stress Drives Dependency on the G2/M Checkpoint in HGSC

### 2.1. CCNE1 Amplification

Oncogene activation can drive replication stress by disrupting the normal coordination of DNA replication and cell cycle progression [16,17]. In HGSC, the most prominent example is amplification of the *CCNE1* gene that encodes the cyclin E1 protein. This genetic alteration occurs in approximately 15–20% of HGSC and is associated with poor outcomes and resistance to standard therapies [18,19,20]. Amplification of *CCNE1* with overexpression of cyclin E1 promotes early CDK2 activation and insufficient loading of replication machinery onto DNA replication origins during the G1 phase, termed origin licensing [21]. This process is important for accurate replication during the S phase. When cyclin E1-CDK2 activity is deregulated, cells may enter the S phase before origins are properly licensed, while also increasing inappropriate origin firing. This dysregulation can lead to replication fork stalling and accumulation of under-replicated DNA. Over time, these defects contribute to replication stress-associated genomic instability [16,22].

### 2.2. HRD-Associated Alterations

Defects in DNA damage repair (DDR) pathways also contribute to replication stress in HGSC. Approximately half of HGSC exhibit homologous recombination deficiency (HRD), most commonly through alterations involving *BRCA1*, *BRCA2*, or other homologous recombination genes [23]. The homologous recombination (HR) repair pathway is largely restricted to the S and G2 phases, when a sister chromatid is available as a repair template. RAD51 recombinase is a central mediator of HR repair, where it forms nucleoprotein filaments on single stranded DNA, followed by identification of a homologous template with subsequent strand invasion and replication. Thus, after DNA damage, the presence of RAD51 nuclear foci is commonly used as a readout of HR activity; reduced RAD51 foci formation may indicate HRD [24]. Cells with unrepaired DNA damage depend on intra-S and G2/M checkpoint signaling to delay cell cycle progression and provide time for HR repair. In HRD tumors, the HR repair capacity is compromised, leading to accumulation of DNA damage as cells progress through the S phase and enter the G2 phase. Under this condition, the G2/M checkpoint becomes extremely important.

The clinical importance of HRD is closely linked to the concept of synthetic lethality. Synthetic lethality occurs when two defects that are individually tolerated become lethal when combined. In HGSC, the best example is the sensitivity of HRD tumors to PARP inhibitors [25]. PARP inhibition impairs repair of DNA lesions and therefore increases DNA damage. In cells with intact HR, this damage can be repaired effectively, but in cells with HR deficiency, the inability to accurately repair DNA breaks leads to the accumulation of lethal damage.

Beyond their role in DNA double strand break repair, BRCA1 and BRCA2 also help stabilize and protect stalled replication forks from nucleolytic degradation [26,27]. Loss of function of these proteins compromises accurate repair of DNA damage and reduces fork protection, allowing DNA damage to accumulate [28]. These defects further increase reliance on checkpoint signaling pathways that preserve replication fork integrity and delay mitotic entry under conditions of genomic stress.

Germline pathogenic variants in other HR and DNA repair genes, including *BRIP1*, *RAD51C*, *RAD51D*, *PALB2*, *BARD1*, and *ATM*, have also been reported in ovarian cancer cohorts [29]. Although these alterations are less common than *TP53* mutation, *CCNE1* amplification, or *BRCA1*/*2* alterations, they support the idea that defects in the maintenance of genome stability can increase replication-associated damage and may strengthen reliance on the G2/M checkpoint.

### 2.3. Other Genetic Alterations

Less common somatic and germline alterations can also contribute to replication stress and subsequent checkpoint reliance in HGSC. TCGA analyses identified recurrent somatic mutations in genes such as *CDK12*, *NF1*, and *RB1*, in addition to *BRCA1*/*2* [10]. These genetic alterations affect different aspects of tumor biology but converge on increased genomic instability, cell-cycle dysregulation, or impaired DNA damage repair. For example, *CDK12* mutations are found in <10% of HGSC [30]; this kinase normally regulates the G2/M checkpoint, and loss of CDK12 creates severe DNA damage repair defects and makes tumors heavily dependent on replication stress signaling [31]. Both *NF1* and *RB1* alterations are found in roughly 10–20% of HGSC [32]. The loss of NF1 leads to upregulation of the mitogen-activated protein kinase (MAPK) and phosphoinositide 3-kinase (PI3K)/AKT stress response pathways, helping cells tolerate DNA damage and stress caused by rapid tumor proliferation [33,34]. The loss of RB1, a core tumor suppressor, deregulates the G1/S checkpoint, which allows uncontrolled replication [35]. In addition to *RB1* genomic alterations, RB pathway dysregulation can also occur through post-translational mechanisms and may indirectly contribute to replication stress by promoting abnormal cell cycle progression. One study reported that RB1 is persistently hyperphosphorylated in HGSC independent of *RB1* copy number alterations, suggesting functional RB inactivation can yield increased proliferation [36].

### 2.4. Replication Fork Instability, Replication-Transcription Conflicts, and Topological Stress

Replication stress can also arise from conflicts between DNA replication and transcription [37]. Collisions between the replication and transcription complexes can stall replication fork progresion, and stalled forks may become unstable, collapse, or generate regions of under-replicated DNA [38,39]. This replication fork instablity increases replication-associated DNA damage and the need for checkpoint signaling to delay cell cycle progression [39].

R-loops can further contribute to replication-transcription conflicts. R-loops are three-stranded nucleic acid structures consisting of an RNA-DNA hybrid and a displaced single-stranded DNA strand. Unresolved or excessive R-loop accumulation can interfere with replication fork movement, increase DNA damage, and promote genomic instability [40,41]. In this way, R-loop-associated replication stress may contribute to replication fork instability and increase reliance on the G2/M checkpoint.

Finally, topological stress is another related contributor, as DNA replication and transcription both generate torsional stress as the DNA helix is unwound [37]. Topoisomerases normally resolve this stress by relaxing DNA supercoiling, allowing replication and transcription to proceed [42]. When topological stress is not properly resolved, replication fork progression can be impaired, and R-loops or replication-transcription conflicts may become more persistent [17,43]. These stresses may further increase the need for checkpoint control to delay cell cycle progression.

### 2.5. Therapy-Induced Replication Stress

Replication stress in HGSC is not only driven by intrinsic genomic alterations but can also be further increased by standard therapies. Platinum-based chemotherapies and PARP inhibitors are cornerstone treatments for HGSC, both targeting defects in DNA repair but through different mechanisms. Platinum-based therapies create DNA lesions that interfere with replication fork progression and require DNA damage repair pathways for resolution [44,45]. PARP enzymes are involved in the repair of DNA single strand breaks and facilitate base excision repair (BER). PARP inhibitors block this repair process, causing unrepaired lesions to persist and interfere with replication. Both treatments increase stress and reliance on the G2/M checkpoint [46], providing a rationale for combining DNA damaging or replication stress-inducing therapies with agents that target checkpoint control.

## 3. Therapeutic Targeting of the G2/M Checkpoint in HGSC

### 3.1. Checkpoint Abrogation

**Figure 2:**
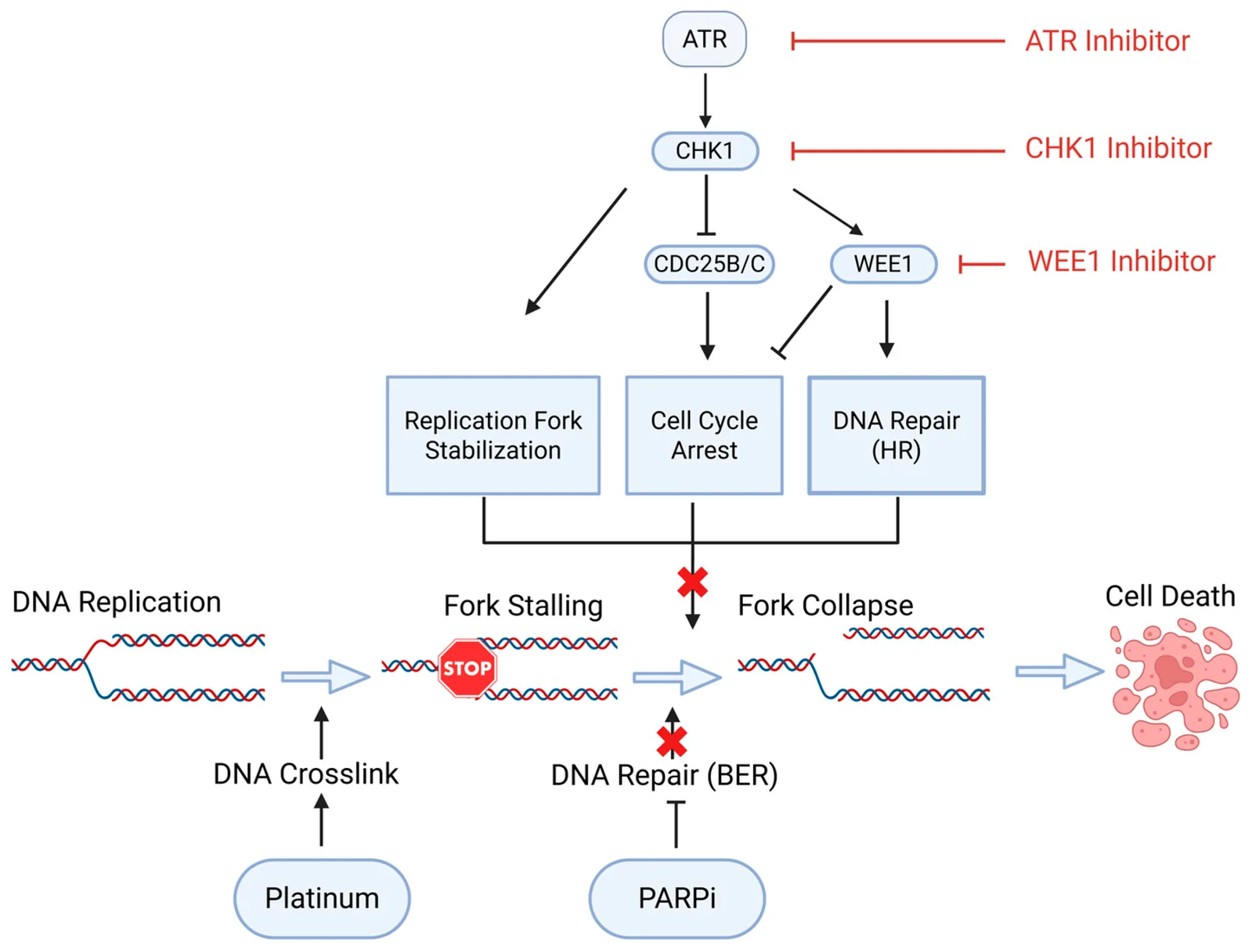
**Checkpoint abrogation under replication stress promotes replication fork collapse and mitotic catastrophe.** Platinum-based chemotherapy and PARP inhibitors (PARPi) increase replication-associated DNA damage through distinct mechanisms. This damage can stall replication forks. Under normal checkpoint control, ATR-CHK1-WEE1 signaling helps stabilize stalled forks, delay cell cycle progression, and allow time for DNA repair. Inhibition of ATR, CHK1, or WEE1 blocks this protective response, leading to unresolved fork stalling and reapplication fork collapse. Fork collapse can lead to additional DNA damage, while release of CDK1-cyclin B inhibition can promote premature mitotic entry. Together, these effects drive mitotic catastrophe and tumor cell death.

A major therapeutic strategy for targeting the G2/M checkpoint is to combine checkpoint inhibition with therapies that increase replication associated DNA damage (Figure 2). Platinum-based agents induce DNA crosslinking that interferes with replication fork progression [44,45], while PARP inhibitors impair repair of DNA lesions [47]. These lesions can stall replication forks and increase dependence on ATR-CHK1-WEE1 signaling to stabilize stalled forks (Figure 2). These kinases normally function to delay mitotic entry under replication stress by maintaining CDK1 in an inhibited state. When ATR, CHK1, or WEE1 activity is blocked, CDK1-cyclin B inhibition is released, promoting premature mitotic entry and leading to mitotic catastrophe (Figure 2).

ATR is a central kinase in the replication stress response. Inhibition of ATR can destabilize stalled replication forks, impair checkpoint control, and promote premature mitotic entry [48]. Several studies have shown that combining ATR inhibition with PARP inhibition can overcome PARP and platinum resistance [49,50,51,52]. Clinically, ceralasertib has been evaluated in combination with olaparib in a phase II trial for recurrent ovarian cancer [50]. In the PARP inhibitor-naïve cohort, the combination was tolerable but did not produce Response Evaluation Criteria in Solid Tumors version 1.1 (RECIST v1.1) objective responses; however, stable disease was observed in nine of 12 evaluable patients, and median progression-free survival (PFS) appeared longer in the subset of patients with *BRCA1*-mutated tumors [50]. These findings suggest that ATR and PARP inhibitor combinations may have limited activity in unselected platinum-resistant disease but could be more relevant in DNA repair-deficient subgroups, which is consistent with the synthetic lethal rationale. However, because the *BRCA1*-mutated subgroup was small, larger studies are needed to determine whether *BRCA1* deficiency is a reliable predictive biomarker for ATR and PARP inhibitor combinations.

CHK1 functions downstream of ATR and coordinates replication checkpoint signaling, replication fork stability, and the HR repair pathway [53,54,55]. Inhibiting CHK1 can impair fork stability resulting in increased replication stress and the accumulation of DNA damage. In ovarian cancer, prexasertib has been the most studied CHK1 inhibitor. Preclinical studies have shown that prexasertib has anti-tumor activity both as monotherapy and in combination with the PARP inhibitor olaparib [56]. Clinical studies have also evaluated prexasertib in recurrent HGSC, including *BRCA*-mutant, *BRCA*-wildtype, platinum-resistant, and PARP inhibitor-resistant contexts [57,58]. In a phase I study of prexasertib plus olaparib, partial responses were observed in a subset of patients with *BRCA1*-mutant, PARP inhibitor-resistant HGSC, with pharmacodynamic evidence of reduced RAD51 foci and increased DNA damage markers after treatment [57]. In a separate phase II study of BRCA-wildtype platinum-resistant HGSC, prexasertib demonstrated single agent activity, although exploratory biomarker analysis suggested that high expression of DNA replication-related genes, including *POLA1*, *POLE*, and *GINS3*, may be associated with lack of clinical benefit [58]. These studies suggest that CHK1 inhibition may be relevant in both PARP inhibitor-resistant *BRCA*-mutant disease and *BRCA*-wildtype platinum-resistant disease, but biomarkers are needed to identify patients most likely to benefit.

WEE1 inhibition provides the most direct connection between CDK1 activation and G2/M checkpoint disruption. WEE1 normally phosphorylates CDK1 at Tyr15 to prevent premature activation of the CDK1-cyclin B complex. When WEE1 is inhibited, CDK1 activity gets promoted and allows cells to enter mitosis despite unrepaired DNA damage, which can lead to mitotic catastrophe. Adavosertib is the best-studied WEE1 inhibitor in ovarian cancer. Clinical studies have evaluated adavosertib in combination with platinum/taxane chemotherapy in *TP53*-mutant ovarian cancer [59], with gemcitabine in platinum-resistant recurrent ovarian cancer [60], and with olaparib in PARP inhibitor-resistant ovarian cancer [61]. In a biomarker enriched phase II trial of *TP53*-mutant platinum-sensitive ovarian cancer, adding adavosertib to carboplatin and paclitaxel improved PFS by enhanced RECIST criteria, although the absolute benefit was modest and higher rates of adverse events were observed [59]. In platinum-resistant or platinum-refractory recurrent HGSC, adavosertib plus gemcitabine significantly improved PFS and overall survival compared with gemcitabine alone [60]. In the PARP inhibitor-resistant setting, a phase II trial showed clinical activity with both adavosertib monotherapy and in combination with olaparib, with response rates of 23% and 29%, respectively [61]. Newer WEE1 inhibitors, such as azenosertib, are also being evaluated in platinum-resistant HGSC [62]. These studies suggest that WEE1 inhibition may be more relevant in tumors with a defective G1 checkpoint, high replication stress, or prior PARP inhibitor exposure.

Recent studies also suggest that the therapeutic consequences of ATR, CHK1, or WEE1 inhibition extend beyond checkpoint abrogation. For example, inhibition of ATR, CHK1, or WEE1 can also serve to induce shift DNA damage repair function resulting in an HRD phenotype, thereby sensitizing tumors to PARP inhibition [63].

### 3.2. Direct CDK1 Inhibition

In contrast to ATR, CHK1, or WEE1 inhibition, which promotes premature mitosis by activating CDK1, direct CDK1 inhibition blocks activation of the CDK1-cyclin B complex and prevents progression from the G2 phase into mitosis, which can result in G2 arrest and reduced tumor cell proliferation. Preclinical studies suggest that CDK1 inhibition can reduce tumor growth in ovarian cancer cells and in a transgenic mouse model [64]. These findings suggest that CDK1 itself may represent a therapeutically relevant node within the G2/M checkpoint network.

However, the consequences of CDK1 inhibition are likely context dependent. Because CDK1 inhibition prevents mitotic entry, it may protect cells from lethal premature mitosis by allowing additional time for DNA repair. Conversely, CDK1 inhibition can also impair CDK1-related DNA damage responses, thereby sensitizing tumor cells to DNA damaging agents. Studies have shown that CDK1 restrains non-homologous end joining (NHEJ) through phosphorylation of NHEJ-related proteins and promotes HR through phosphorylation of HR-related proteins, indicating that CDK1 inhibition has effects beyond cell cycle regulation [65,66,67,68,69]. These findings suggest that CDK1 inhibition may affect therapeutic response not only by blocking mitotic entry, but also by altering DDR pathway utilization. However, the relevance of this mechanism to HGSC requires further study. Since checkpoint abrogation often depends on CDK1 activation to force premature mitosis, direct CDK1 inhibition could theoretically blunt this effect if given at the wrong time. This highlights the importance of treatment timing and mechanistic context.

### 3.3. Additional Regulators of CDK1 Activation

**Figure 3:**
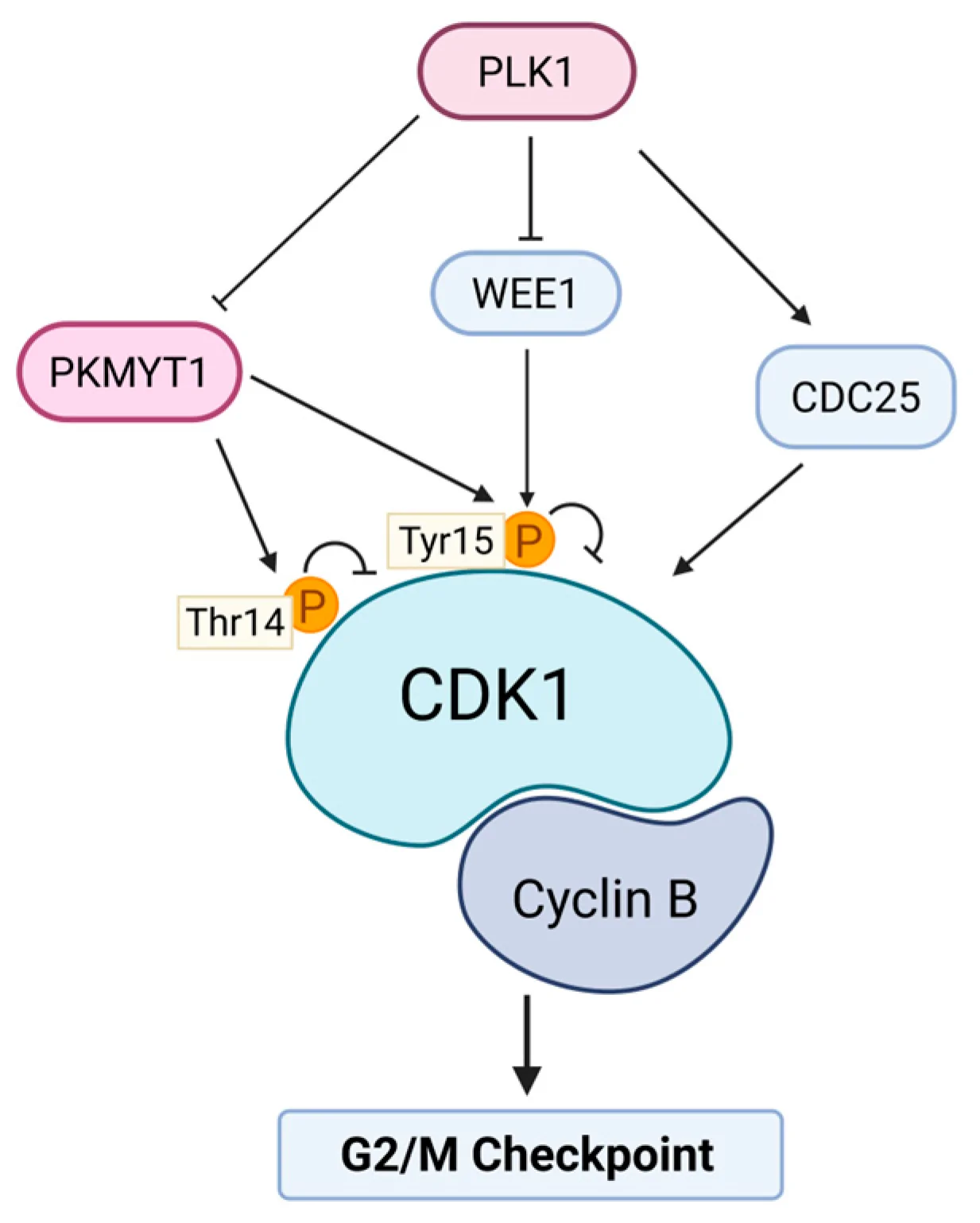
**Regulation of CDK1-cyclin B activity by PKMYT1 and PLK1.** CDK1-cyclin B activation is controlled by the balance between inhibitory phosphorylation and activating dephosphorylation. PKMYT1 phosphorylates CDK1 at Thr14 and Tyr15, while WEE1 also phosphorylates CDK1 at Tyr15, maintaining CDK1-cyclin B in an inhibited state and restraining premature mitotic entry. CDC25 phosphatases remove inhibitory phosphorylation from CDK1 to promote CDK1-cyclin B activation. PLK1 reinforces mitotic entry by promoting CDC25 activity and contributing to WEE1 degradation.

Since CDK1 activation is controlled by multiple upstream regulators, therapeutic targeting of the G2/M checkpoint may extend beyond ATR, CHK1, WEE1, and CDK1 alone. Protein kinase membrane-associated tyrosine (PKMYT1) and polo-like kinase 1 (PLK1) are two regulators that influence the balance between CDK1 inhibition and activation (Figure 3).

PKMYT1 inhibits CDK1 through phosphorylation at Thr14, complementing WEE1-mediated inhibitory phosphorylation at Tyr15 [70], whereas CDC25 phosphatases remove this inhibitory phosphorylation to promote CDK1-cyclin B activation [71]. PLK1 further shifts this balance toward mitotic entry by promoting CDC25 activity and contributing to WEE1 degradation [72] (Figure 3). In tumors that have high replication stress, PKMYT1 may help restrain premature mitosis. A recent study demonstrates that combined inhibition of PKMYT1 and ATR promotes CDK1 activation, forcing premature mitotic entry and inducing mitotic catastrophe [22].

PLK1 acts in the opposite direction by also promoting CDK1 activation. Mechanistically, PLK1 can support CDK1-cyclin B activation by promoting CDC25 activity and contributing to WEE1 degradation [70,73]. Rather than functioning as a DNA damage checkpoint kinase like ATR or CHK1, PLK1 acts mainly as a mitotic entry kinase that reinforces CDK1-cyclin B activation [74]. This creates a different therapeutic logic from PKMYT1 inhibition. Whereas PKMYT1 inhibition releases inhibitory phosphorylation on CDK1 and forces premature mitotic entry, PLK1 inhibition interferes with the machinery required for mitotic entry. Because PLK1 is required for several mitotic processes, including centrosome maturation, spindle formation, chromosome segregation, and cytokinesis, inhibiting PLK1 can cause G2/M arrest, abnormal mitosis, DNA damage accumulation, and apoptosis [75]. The selective PLK1 inhibitor onvansertib combined with olaparib had an anti-tumor effect in olaparib-resistant HGSC models, including *BRCA1*-mutant, *BRCA1*-wildtype models and patient-derived xenografts [76]. A randomized phase II study investigated the PLK1 inhibitor volasetib versus single-agent chemotherapy in ovarian cancer patients with platinum-resistant or platinum-refractory disease and found a 24-week disease control rate of 30.6% for volasertib and a 43.1% rate for chemotherapy, with manageable adverse events [77].

### 3.4. Clinical Translation of G2/M Checkpoint Targeted Therapies

Several components of the G2/M checkpoint pathway have entered clinical evaluation in ovarian cancer, particularly in recurrent, platinum-resistant, or PARP inhibitor-resistant disease settings. Current clinical strategies most commonly evaluate ATR, CHK1, or WEE1 inhibitors, either as single agents or in combination with DNA damaging and replication stress-inducing therapies such as platinum agents, gemcitabine, or PARP inhibitors. Additionally, multiple clinical trials are investigating the efficacy of targeting the G1/S phase transition of the cell cycle, including small molecule inhibitors of the CDKs and ATM. Ongoing clinical trials targeting the G2/M checkpoint pathway in HGSC are summarized in Table 1. Mechanistically, each of these targets disrupts the G2/M checkpoint. ATR inhibitors block the normal upstream signaling that is required to pause the cell cycle (Figure 2) which compounds DNA damage. CHK1 inhibitors block CHK1-mediated cell cycle arrest and WEE1 inhibitors prevent its G2/M gatekeeper function resulting in damaged cells proceeding past the checkpoint (Figure 2). Finally, PKMYT1 inhibition prevents CDK1 regulation (Figure 3) which forces cancer cells into early mitosis before completing DNA replication.

Together, these clinical trials highlight several key points to note in the clinical development of G2/M checkpoint targeted therapy. First, this overall strategy of targeting these proteins is a relatively new strategic approach. Second, biomarker selection remains an important factor, with biomarkers an inclusion criterion for some of the trials. As discussed further below, the integration of biomarkers into both trials and patient selection for therapy will be essential. Genomic features may help identify tumors that are more likely to depend on the G2/M checkpoint, but predictive biomarkers are still being refined.

## 4. Conclusions and Future Directions

Persistent replication stress with loss of the G1 checkpoint results in an increased reliance on the G2/M checkpoint in tubo-ovarian HGSC. This dependency represents a potentially exploitable therapeutic vulnerability and provides a mechanistic framework for the development of targeted therapies, particularly in the combinatorial treatment setting. By disrupting key regulators of mitotic entry, such as ATR, CHK1, WEE1, and CDK1, tumor cells may be forced to enter mitosis with unresolved DNA damage, leading to mitotic catastrophe. We feel that targeting the G2/M checkpoint is a clinically relevant opportunity in the treatment of HGSC. There are several factors that are required for successful clinical translation.

First, the continued integration of biomarkers into both preclinical studies and clinical trials is needed to identify tumors most likely to respond to G2/M checkpoint targeted therapies. Biomarkers currently being investigated include *TP53* mutations, Cyclin E overexpression and/or *CCNE1* amplification, BRCA and/or HRD status, replication stress signatures, and functional readouts of checkpoint activation such as elevated γH2AX. As precision medicine evolves for all cancer therapies, we strongly believe that biomarker-driven treatment selection will improve outcomes.

Second, optimal combination strategies need to be further defined and then explored in preclinical and clinical settings. Because platinum-based agents and PARP inhibitors increase DNA damage, combining these agents with checkpoint inhibitors may enhance therapeutic response, but treatment timing and sequencing will likely be critical. Additionally, a major potential limitation of checkpoint-directed therapy is toxicity, particularly if used in combination with other agents.

Third, it will be important to understand how tumor cells adapt to checkpoint-targeted therapies and whether these adaptive responses differ across underlying genetic backgrounds. Overall, the G2/M checkpoint should be viewed as a survival pathway in high replication stress HGSC. As the use of checkpoint inhibitors evolves, the subsequent resistance mechanisms and potential for overcoming those mechanisms will be an important component of clinical translation.

In conclusion, HGSC is a disease in need of new treatment approaches to improve clinical outcomes. Targeting the G2/M checkpoint is a potential treatment strategy that exploits the underlying biology of the disease and may provide clinically meaningful responses in at least a subset of biomarker-selected patients. A deeper understanding of how HGSC cells regulate mitotic entry under stress may guide biomarker development, improve combinatorial treatment strategies, and support the clinical translation of G2/M checkpoint targeted therapies.

## Figures and Tables

**Table 1:** Current clinical trials in patients with ovarian, fallopian tube, and primary peritoneal cancers using agents that target proteins regulating the G2/M checkpoint.

Target	Agent(s)	Cancer Types	Biomarker Inclusion Criteria	Phase	Status	Sponsor	Trial Number
ATR	M1774 (ATR inhibitor) plus ZEN-3694 (BET^†^ inhibitor)	Ovarian, endometrial	Part II stratified by *ARID1A* status	Ib	Recruiting	National Cancer Institute	NCT05950464
ATR	Ceralaertib or 14C-Ceralasertib	Ovarian, endometrial, NSCLC	--	I	Not yet recruiting	AstraZeneca	NCT06754761
ATR	ART0380 alone and plus chemotherapy	Solid tumors including ovarian	Part B1/B5/B6 require *ATM* alteration	I/IIa	Recruiting	Artios Pharma, Ltd.	NCT04657068
CHK1	XS-02	Phase I—solid tumors including ovarian cancer; phase II—HGSC	--	I/II	Not yet recruiting	NovaOnco Therapeutics Co., Ltd.	NCT06531486
WEE1	Azenosertib (ZN-c3)	HGSC	Positive Cyclin E1 protein status	II	Recruiting	Zentalis Pharmaceuticals, Inc.	NCT05128825
PKMYT1	RP-6306 plus chemotherapy	Ovarian, uterine	*TP53* mutation	I	Active, not recruiting	University Health Network, Toronto	NCT06107868

## Data Availability

No new data were created or analyzed in this study.

## Abbreviations

The following abbreviations are used in this manuscript:

ATM	Ataxia-telangiectasia mutated
ATR	Ataxia telangiectasia and Rad3-related
ATRIP	ATR-interacting protein
BET	Bromodomain and extra-terminal
BER	Base excision repair
CDK	Cyclin-dependent kinase
CHK	Checkpoint kinase
DDR	DNA damage repair
E2F	E2 promoter-binding factor
HGSC	High-grade serous carcinoma
HRD	Homologous recombination deficiency
HR	Homologous recombination
MAPK	Mitogen-activated protein kinase
NHEJ	Non-homologous end joining
PARP	Poly (ADP-ribose) polymerase
PFS	Progression-free survival
PI3K	Phosphoinositide 3-kinase
PKMYT1	Protein kinase membrane-associated tyrosine
PLK1	Polo-like kinase 1
RB	Retinoblastoma protein
RECIST	Response Evaluation Criteria in Solid Tumors
RPA	Replication protein A
